# Comparative evaluation of cerebral gliomas using rCBV measurements during sequential acquisition of T1-perfusion and T2*-perfusion MRI

**DOI:** 10.1371/journal.pone.0215400

**Published:** 2019-04-24

**Authors:** Jitender Saini, Rakesh Kumar Gupta, Manoj Kumar, Anup Singh, Indrajit Saha, Vani Santosh, Manish Beniwal, Thennarasu Kandavel, Marc Van Cauteren

**Affiliations:** 1 Department of Neuroimaging & Interventional Radiology, National Institute of Mental, Health and Neurosciences, Bangalore, Karnataka, India; 2 Department of Radiology and Imaging, Fortis Memorial Hospital and Research Institute, Gurgaon, Haryana, India; 3 Center for Biomedical Engineering, Indian Institute of Technology Delhi, Hauz Khas, New Delhi, India; 4 Philips Health Systems, Philips India Limited, Gurgaon, Haryana, India; 5 Department of Neuropathology, National Institute of Mental, Health and Neurosciences, Bangalore, Karnataka, India; 6 Department of Neurosurgery, National Institute of Mental, Health and Neurosciences, Bangalore, Karnataka, India; 7 Department of Biostatistics, National Institute of Mental, Health and Neurosciences, Bangalore, Karnataka, India; 8 Philips Health Tech Asia Pacific, Minato-ku Tokyo, Japan; Henry Ford Health System, UNITED STATES

## Abstract

**Objective:**

To assess the inter-technique agreement of relative cerebral blood volume (rCBV) measurements obtained using T1- and T2*-perfusion MRI on 3T scanner in glioma patients.

**Methods:**

A total of 49 adult patients with gliomas underwent both on T1- and T2*-perfusion in the same scanning session, and rCBV maps were estimated using both methods. For the quantitative analysis; Two independent observers recorded the rCBV values from the tumor as well as contralateral brain tissue from both T1- and T2*-perfusion. Inter-observer and inter-technique rCBV measurement agreement were determined by using 95% Bland-Altman limits of agreement and intra-class correlation coefficient (ICC) statistics.

**Results:**

Qualitative analysis of the conventional and perfusion images showed that 16/49 (32.65%) tumors showed high susceptibility, and in these patients T2*-perfusion maps were suboptimal. Bland-Altman plots revealed an agreement between two independent observers recorded rCBV values for both T1- and T2*-perfusion. The ICC demonstrated strong agreement between rCBV values recorded by two observers for both T2* (ICC = 0.96, p = 0.040) and T1 (ICC = 0.97, p = 0.026) perfusion and similarly, good agreement was noted between rCBV estimated using two methods (ICC = 0.74, P<0.001). ROC analysis showed that rCBV estimated using T1- and T2*-perfusion methods were able to discriminate between grade-III and grade-IV tumors with AUC of 0.723 and 0.767 respectively. Comparison of AUC values of two ROC curves did not show any significant difference.

**Conclusions:**

In the current study, T1- and T2*-perfusion showed similar diagnostic performance for discrimination of grade III and grade IV gliomas; however, T1-perfusion was found to be better for the evaluation of tumors with intratumoral hemorrhage, postoperative recurrent tumors, and lesions near skull base. We conclude that T1-perfusion MRI with a single dose of contrast could be used as an alternative to T2*-perfusion to overcome the issues associated with this technique in brain tumors for reliable perfusion quantification.

## Introduction

Perfusion-MRI is used in the daily clinical practice for the evaluation of hemodynamic changes in brain tumors for more than a decade and serves as a surrogate marker for tumor angiogenesis [1,2]. Two most common techniques used for contrast-enhanced perfusion-MRI are; a T2*-based dynamic susceptibility contrast (DSC) perfusion MRI, and T1-based dynamic contrast-enhanced (DCE) MRI. In DCE/T1-perfusion, contrast causes an increase in signal intensity proportional to the tissue contrast concentration, while in DSC/T2*-perfusion, first pass of gadolinium-based contrast material through the vasculature causes signal drop due to the susceptibility effects caused by contrast bolus [3–5]. A relative cerebral blood volume (rCBV) map, which provides a measure of the tumor neovascularization, is the most widely used quantitative hemodynamic perfusion metrics [6]. The rCBV maps serve as an important imaging biomarker and extensively used for brain tumor grading, post-surgical tumor assessment, and treatment response [5,7,8].

T2*-perfusion commonly uses single-shot gradient echo based echo planar imaging (EPI) sequence which is highly sensitive to susceptibility as it aims to detect small signal loss due to a contrast bolus [5]. However, excessive sensitivity to susceptibility makes perfusion estimation difficult and erroneous in the presence of blood product, calcifications, proximity to aerated sinuses, adjacent bony structures, lesions close to the skull base and postsurgical lesion evaluation, which are commonly encountered in the clinical brain tumor imaging [9,10]. For T2*-perfusion, the quantification of cerebral perfusion usually relies on the assumption of an intact blood-brain barrier (BBB) which is generally not true in case of brain tumors where BBB is disrupted [11]. The assumption of intact BBB is known to be a significant source of error, leading to an inaccurate estimation of rCBV values. Despite these limitations, T2*-perfusion has been used for the preoperative evaluation, assessment of treatment response and differentiation of tumor recurrence and radiation necrosis in gliomas [6–8,12,13].

On the other hand, T1-perfusion is less susceptible to the presence of blood products including hemorrhage, calcification, and artifacts associated with postsurgical changes and expected to give a more accurate assessment of the lesions associated with these issues. [14,15]. In T1-Perfusion, the effect of leakage on rCBV is mitigated by using tracer-kinetic parameters [16,17]. Though T1-perfusion has a low temporal resolution for estimation of hemodynamic parameters, several studies have shown that rCBV estimation using T1-perfusion method is feasible and can also be estimated accurately with if sufficient temporal resolution is present [18,19]. T1-perfusion derived hemodynamic and kinetic parameters have been found useful for predicting disease progression, therapy assessment, and survival in brain tumor patients [20,21].

Despite, the availability of these advanced perfusion-MRI methods in a clinical setting for brain tumor diagnosis and treatment assessment for more than decades, the systematic comparison of these two approaches for evaluation of brain tumors is relatively scarce. Only a few studies have used both the techniques for assessment of gliomas, and very rarely hemodynamic metrics derived from both T1- and T2*-perfusion have been compared [20,22–25].

This study aimed to compare the performance of T1- and T2*-perfusion MRI for the evaluation of glial neoplasm and also to evaluate whether T1-perfusion could be a valid alternative to the T2*-perfusion for the quantitative evaluation of brain tumor in a routine clinical setting.

## Materials and methods

### Patient selection

The National Institute of Mental Health and Neurosciences, Bangalore, Karnataka, India, Institutional Ethics Committee approved this study protocol. A retrospective review of hospital records and PACS revealed a total of 58 adult patients with histopathologically proven gliomas who had undergone both T1- and T2*-perfusion MRI as a part of brain tumor evaluation. The informed consent was waived off for by ethics committee for this retrospective study. Nine patients were excluded from the final analysis due to incomplete or poor perfusion data quality. Hence, the final data analysis was performed in 49 cases, these patients presented with symptoms related to a space-occupying intracranial mass lesion.

### MR imaging protocol

All patients underwent conventional and perfusion-MRI (T1- and T2*-) in the same scanning session on 3.0T MRI (Achieva, Philips Health Tech, The Netherlands) using 32-channel head-coil. MR imaging protocol included the following sequences: T1-weighted images [TR/TE = 2,000/20ms, inversion time (TI) = 800ms, number of slices = 28, slice-thickness = 5mm, FOV = 230×184mm^2^, matrix = 400x272]; T2-weighted images [TR/TE = 3,500/110ms, slice-thickness = 4mm, matrix = 384×384, FOV = 230×230mm^2^]. The acquisition parameters for the 3D FLAIR imaging were the following [TR/TE/TI = 4,700/288/1,650ms, refocusing -angle = 90°, slice thickness = 0.56mm over contiguous slices, matrix = 204×204, FOV = 230×184mm^2^]; T2-weighted GRE [TR/TE = 850/16ms, flip-angle = 18; slice-thickness = 4 mm, matrix = 256×163, FOV = 240x183mm^2^]; Susceptibility weighted imaging (SWI) [TR/TE = 46/25ms, flip-angle = 15°, slice-thickness = 1.0mm, matrix = 232×178, FOV = 230×180mm^2^]; respectively.

Initially, T1-perfusion data was acquired using a 3D turbo field echo (TFE) sequence; (TR/TE = 4.4/2.1ms, flip-angle = 10^0^, slice-thickness = 6.0mm, FOV = 240×240mm^2^, matrix = 128×128, dynamics = 32, slices = 12, with temporal resolution of 3.9s), followed by T2*-perfusion using a gradient echo T2*-weighted EPI sequence (TR/TE = 1574/40ms, slices = 24; flip-angle = 75^0^, FOV = 240x240mm; slice-thickness = 4.0mm; matrix = 96x96 with temporal-resolution of 1.6s). For both perfusion-MRI examinations, gadolinium-diethylenetriamine Penta-acetic acid (Gd-DTPA, Omniscan, GE Healthcare, Milwaukee, USA), was administered intravenously (0.1mmol/kg) as a bolus through an autoinjector (Medrad Spectris Solaris EP MR Injection System, Bayer HealthCare, USA) after 3^rd^ dynamic in T1-based (11.7s), and 10^th^ dynamic in T2*- (16s) at the rate of 3.0ml/sec, and 5.0ml/sec respectively, followed by a bolus injection of 30ml of the physiological saline solution at each time. Contrast agent leakage through BBB breakdown can reduce T2* signal decay because of T1 shine effect related signal increase in extravascular space. Therefore, the rationale for the performing T1-perfusion before T2*-perfusion was to preload contrast agent during the T1-perfusion which can reduce the T1 shine effect and T2* contrast agent leakage effects on the measurements of rCBV from the T2*-perfusion MRI [24,26].

### Image evaluation, processing, and data analysis

Two radiologists (RKG and JS with 30 and 15 years of experience in neuroradiology; respectively) blinded to clinical and histopathology results independently evaluated the conventional MRI data on a PACS workstation.

### Qualitative evaluation

Conventional MRI and rCBV maps were visually inspected to look for the presence of susceptibility within the lesions or arising due to the proximity of the lesion to the skull base or in the vicinity of the paranasal sinuses. Both reviewers individually analyzed the images and saw if the perfusion evaluation of the whole tumor could be accomplished or only partial evaluation was possible.

### Quantitative analysis

T2*-perfusion data was analyzed, and rCBV maps were computed using vendor supplied Intellispace Portal 9.0 (Philips Health Tech), which included motion correction, and registration followed by a manual AIF selection, and deconvolution. rCBV maps generated from T2*-perfusion were further assessed in the Intellispace portal, and multiple ROIs are ranging from 3–6 with each measuring 5–10 mm in diameter was drawn on the rCBV map and selecting the regions showing the highest value of the CBV within the tumor from each patient. While recording the rCBV values care was taken to avoid placement of ROI on large vessels and areas with frank susceptibility. For this purpose, the rCBV maps were coregistered with FLAIR/T2-weighted, post-contrast T1-weighted or SWI/GRE images and thus avoiding inappropriate placement of the ROI. Contra-lateral ROIs were placed on the healthy brain white matter avoiding vessels to obtain the normalized rCBV values. The parts of the tumor showing susceptibility artifacts on gradient/SWI images were not used for placement of ROIs.

All T1-perfusion data were transferred to an offline workstation dedicated to post-processing and quantification. T1-perfusion data were registered concerning precontrast T1-weighted images. T1-perfusion data were analyzed using an in-house developed software [16,27]. The pre-contrast T_1_ map was computed using a previously described method based on T_1_, T_2,_ and PD-weighted TSE images [16]. After pre-contrast T1 estimation, voxel-wise signal intensity time curves obtained from T1-perfusion were converted to concentration-time curves using the procedure described previously [16,17]. These voxel-wise concentration curves were fitted to the LTKM model for the estimation of various parameters as described by Sahoo et al. [27]. Hemodynamic parameters (relative CBV, and CBF) were estimated by first-pass analysis of concentration-time curves [16]. CBV map was also corrected for leakage (CBV_corr) of contrast using a previously reported method [16]. The same strategy was followed for correct ROI placement as described above for the T2*-perfusion. ROIs were placed within the tumor as well as contralateral white matter using Image-J tool, multiple ROIs measuring 5–10 mm were placed avoiding large vessels or veins by co-registering the rCBV maps with the FLAIR/T2-weighted, post-contrast T1-weighted, and SWI-images. The rCBV value was also recorded from the contralateral normal brain parenchyma in order to calculate the normalize rCBV. For the final statistical analysis, we used the normalized rCBV values from both T1- and T2*- perfusion data.

### Statistical analysis

The variables were tested for normal distribution. The maximal rCBV values for both T2*- and T1-perfusion images recorded by two observers were compared using a paired sample test. For T2*- and T1-perfusion derived rCBV values recorded by the two observers, inter-observer agreement was assessed using Intraclass correlation coefficient (ICC), which is categorized as the ICC<0.40 = poor, 0.40–0.59 = fair, 0.60–0.74 = good, and ICC>0.74 or above = excellent [28].

Variance in the values recorded by two observers was also assessed by Pitman’s test of difference in variance. Also, Bland-Altman plots were constructed to look for “agreement limits” between the rCBV values dervied from the two modality (T2* and T1- perfusion). We also looked for agreement limits between the rCBV values recorded by two independent observers. If this interval presents a wide range of variation, it is supposed that there is no concordance between the two measurements and the reproducibility hypothesis is rejected. A visual inspection of the graph shows the data dispersion. If there is a concordance between two measures, the different values scatter along the zero line. The relationship between the T2*- and T1- derived rCBV was computed using Spearman non-parametric correlation.

Receiver operating characteristic (ROC) curve analysis was used to evaluate the performance of rCBV individually in discriminating between grade-III and grade-IV gliomas by comparing the area under the curve (AUC). The sensitivity and specificity were established to discriminate between grade-III and grade-IV gliomas.

All the statistical computations were carried out using Statistical Package for Social Sciences (SPSS, version 22.0, Armonk, NY: IBM Corp. and R Stats Package software version 3.5.0, Boston, MA, USA). A p-value of ≤0.05 was considered statistically significant.

## Results

A total of 49 patients with supratentorial gliomas were included in this study. All patients were histopathologically confirmed and classified according to the latest WHO classification [29]. Six patients had low-grade lesions; thirty-one had grade-III, and twelve harbored grade-IV tumors (Table 1). Out of 49 cases, there were 6 cases with post-surgery tumor recurrence.

**Table 1:** Patient demographic lesion characteristics and lesion locations.

S. No.	Clinical parameters	Measures (number)
**1.**	**Total number**	N = 49
**2.**	**M: F**	31:18
**3.**	**Mean age (in the year)**	39.78 (18–84)
**4.**	**Grade I and II (n = 6)**	Pilocytic (2), Pilomyxoid astrocytoma (1), Astrocytoma (2), Oligodendroglioma (1)
	**Grade III (n = 31)**	Anaplastic Oligodendroglioma (11), Anaplastic Astrocytoma (10), Anaplastic Oligoastrocytoma (5) Anaplastic ependymoma (5)
	**Grade IV (n = 12)**	Glioblastoma (12)
**5.**	**Lesion location**	
	**Corpus callosum**	2
	**Basal Ganglia**	2
	**Thalamus**	5
	**Insular**	5
	**Frontal**	12
	**Temporal**	7
	**Parietal**	10
	**Occipital**	2
	**Cerebellum**	4

### Qualitative analysis

**Fig 1:**
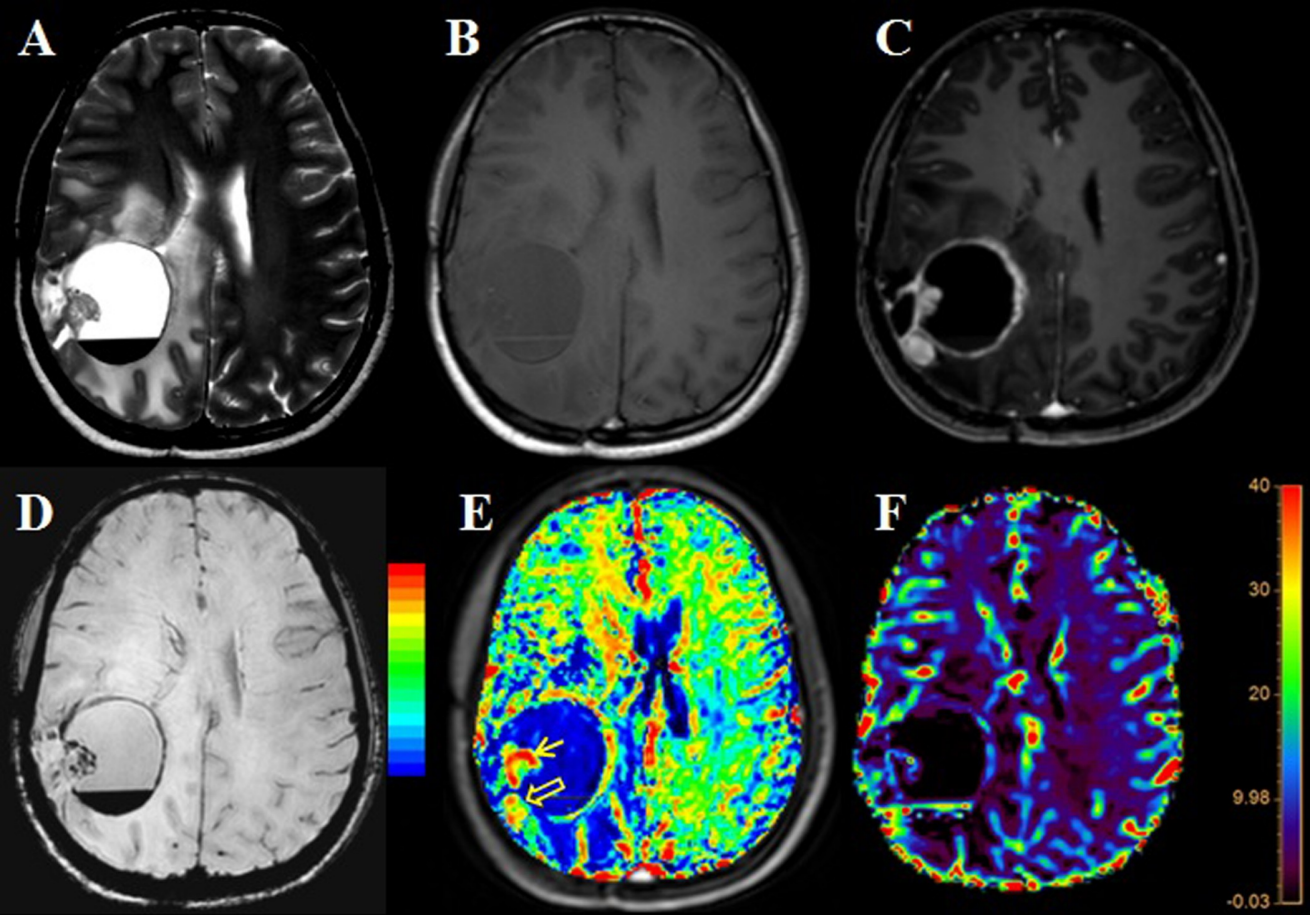
A post-operative case of high-grade glioma showing recurrent tumor with foci of susceptibility in the mural nodule as well as blood fluid level in the cystic component (E, open arrow). The mural nodule is showing high CBV on T1- perfusion (E, thick arrow). The corresponding area shows relatively low CBV on T2* perfusion MRI. Another foci with increased CBV seen posteriorly detected well on both T2*- and T1- perfusion MRI (E and F, thin arrows). Artifactual increased CBV is seen within the cystic component of the tumor on T2*- perfusion (F, thin arrow).

**Fig 2:**
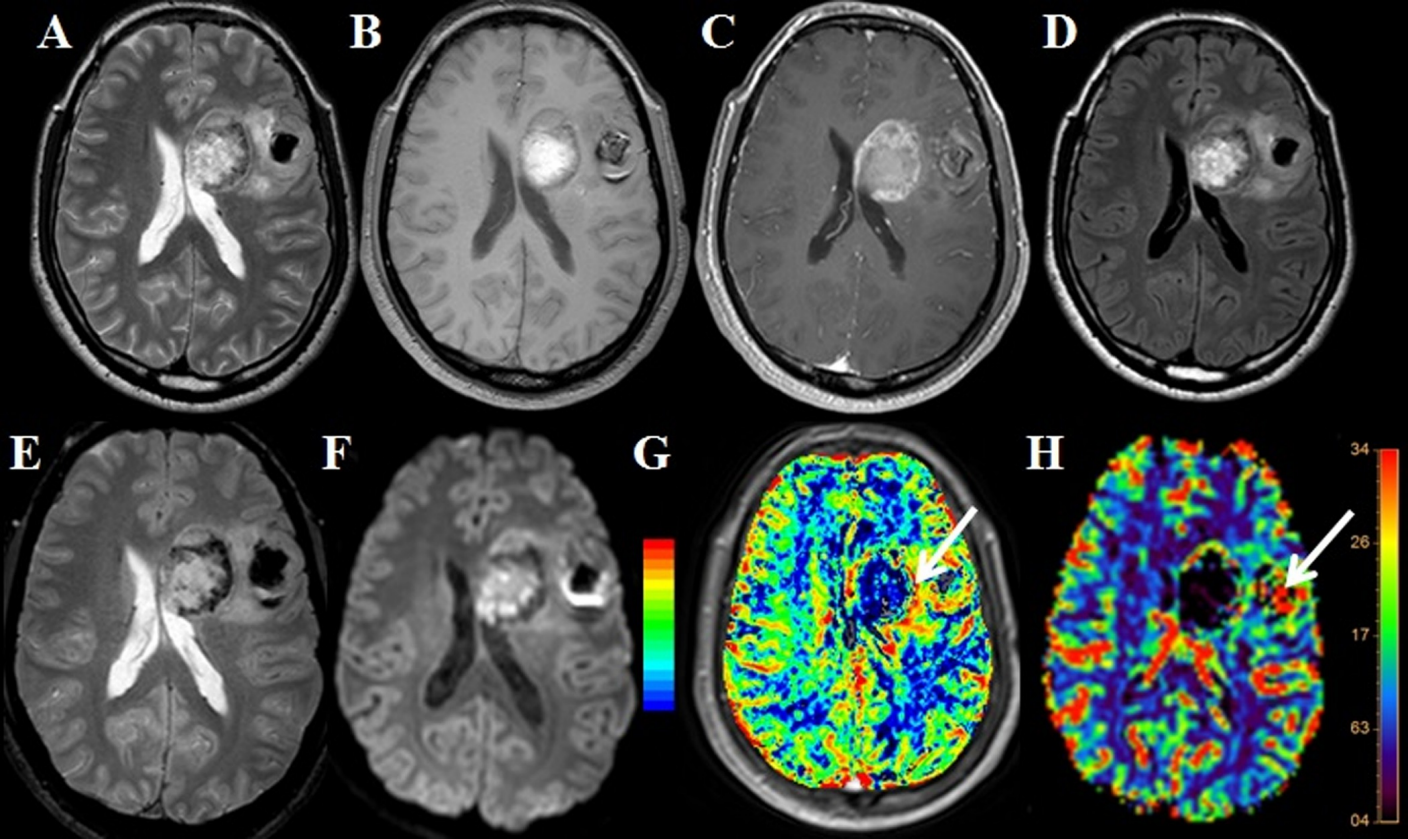
Gliomas are showing abundant susceptibility on gradient imaging: T1- perfusion showing the peripheral area of the increase rCBV (arrow). T1- perfusion also shows the scattered area of increase perfusion. On T2*-perfusion subtle increase in rCBV noted, and artificially high rCBV perfusion was seen in the part of tumor showing susceptibility.

On T2*-perfusion, 16/49 (32.65%) patient’s evaluation was suboptimal due to the presence of the high susceptibility artifacts arising due to various reasons and entire tumor could not be evaluated on T2*-perfusion data from these patients; however, all these cases could be adequately evaluated by using T1-perfusion. Geometrical distortions and signal loss causing marked image degradation on the T2*-perfusion corresponding to FLAIR signal abnormality were considered as suboptimal T2*-perfusion. Such lesions influenced by susceptibility image degradation and had a poor diagnostic value of the rCBV maps derived from the T2*-perfusion, and tumor evaluation was reported suboptimal or difficult by both reviewers. In these patients, the highest rCBV values were recorded from the parts of the tumor which were not affected by the presence of susceptibility. Fig 1 demonstrates an example of poor T2*-perfusion from one of the subjects from this study. Various causes of suboptimal T2*-perfusion data in these sixteen subjects were the presence of the intratumoral susceptibility due to hemorrhage (n = 7), post-surgery signal susceptibility (n = 4), lesions located near the skull base or sinuses (n = 5) which precluded the adequate evaluation. Fig 2 represents a typical T1- and T2*- perfusion characteristics of the postsurgical tumor.

### Quantitative analysis

On both T1- and T2*- perfusion, there was no significant difference in the maximum rCBV values recorded by two observers as well as between two methods. The ICC with a 95% confidence interval for interobserver agreement on T2*-perfusion was excellent measuring 0.96 for maximum rCBV values (p = 0.040) (Table 2). The means of the maximum rCBV values recorded by two observers on T2*-perfusion derived rCBV maps were 5.438±4.410 and 4.956±3.050, respectively. Pitman's test was positive and significant differences in the variance of T2*-perfusion measured rCBV recorded by two observers was noted.

**Table 2:** Summary of T1- and T2*-perfusion derived rCBV values discriminating grade—III (n = 31) and grade -IV (n = 12) astrocytic brain tumors.

rCBV values	Grade III (mean±SD)	Grade IV (mean±SD)	P-value	AUC	ICC-AUC	Cut-off	Sensi-tivity	Speci-ficity	P-value
T2*-rCBV	3.954±2.41	8.485±6.19	0.037	0.764	0.96	4.737	81%	72%	0.040
T1- rCBV	4.790±2.63	7.364±3.45	0.041	0.727	0.97	5.480	78%	63%	0.026

AUC = Area under curve, T2*-perfusion MRI (Dynamic susceptibility contrast); T1-perfusion MRI (Dynamic contrast enhanced); ICC = Intraclass correlation coefficients; rCBV = Relative cerebral blood volume.

Note: T1- and T2*-perfusion MRI derived mean rCBV values reported in the table are taken from observer one.

**Fig 3:**
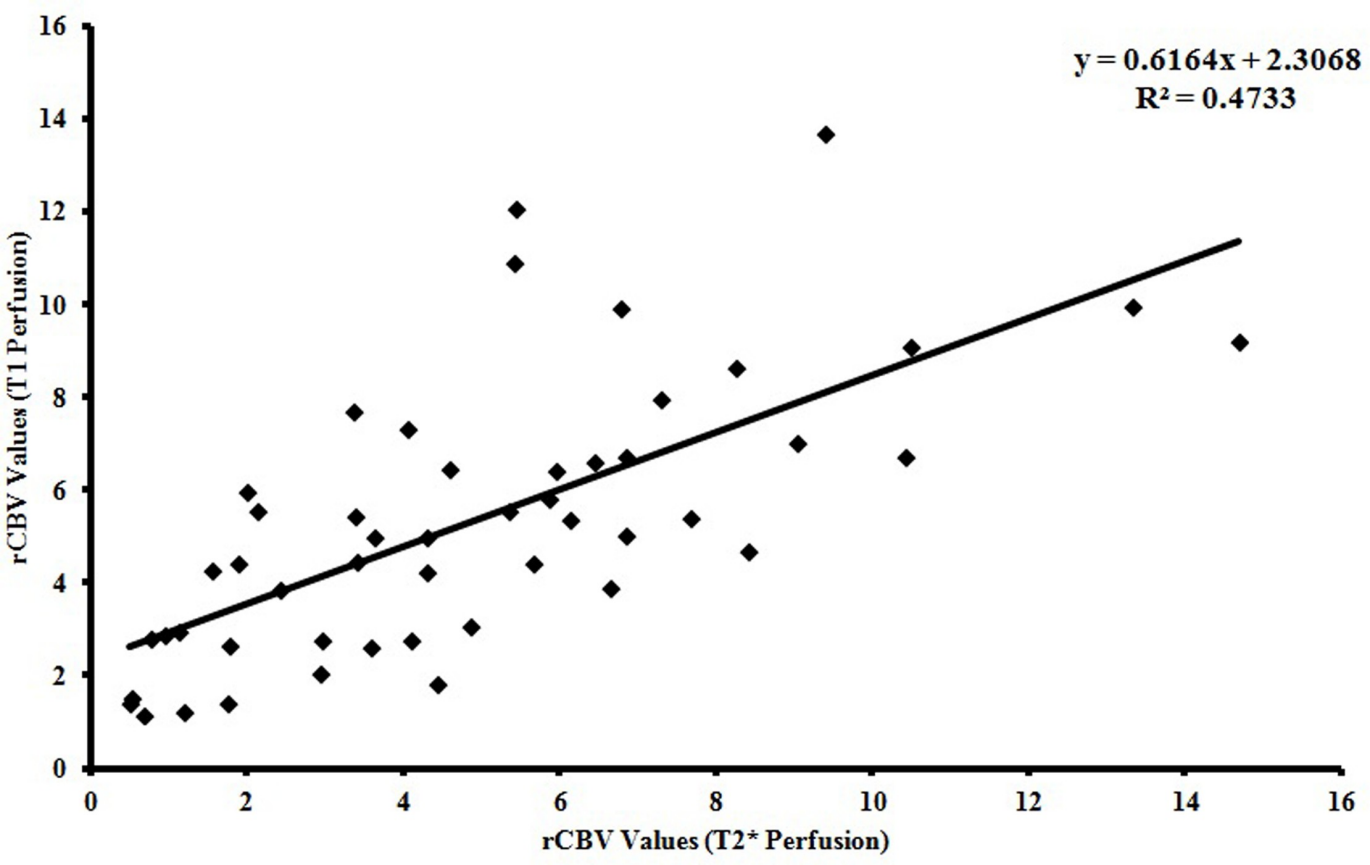
Scatterplot of rCBV values derived from T1- and T2*-perfusion MRI data demonstrating the distribution of the rCBV values recorded by these two methods.

**Fig 4:**
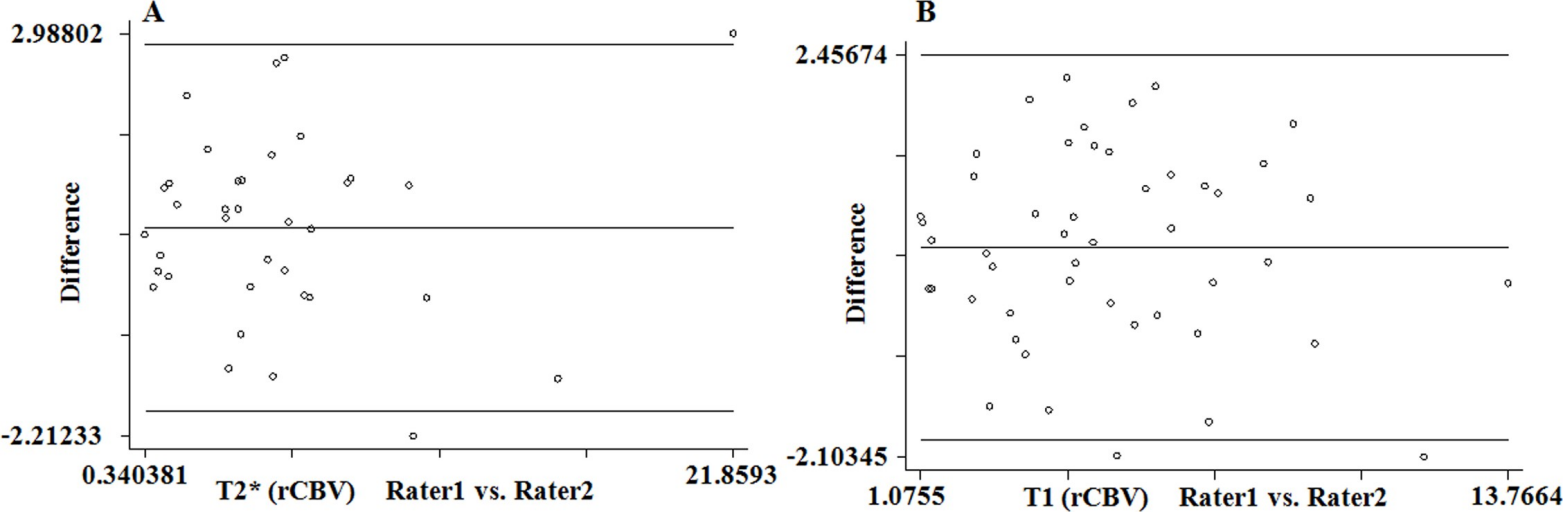
Bland-Altman is demonstrating the limits of agreements between two independent observers recorded the rCBV values from T2*- and T1- perfusion MRI data. The midline represents the mean values for the data points, and the top and bottom line represents the 1.96 and -1.96 limits of agreements, respectively.

The ICC with a 95% confidence interval for interobserver agreement on T1-perfusion was an excellent measuring 0.97 for maximum rCBV (p<0.001) (Table 2). The means of the maximum rCBV values recorded by two observers on T1-perfusion was 5.270±2.831 and 4.994±2.918, respectively. No significant difference in the variance was noted between T1-perfusion measured rCBV recorded by two readers. The scatterplot of rCBV values derived from T1- and T2* perfusion MRI were also plotted to evaluate the distribution of the rCBV values recorded by these two methods. The scatterplot demonstrate the agreement between these two method with no statistically significant difference between these two methods (Fig 3; R^2^ = 0.4733). The Bland-Altman plots also showed good agreement between the rCBV values recorded by two independent observers for measuring rCBV derived from T2*- and T1-perfusion methods; respectively (Fig 4). A significant correlation was also noted in the rCBV values calculated using these two methods (r = 0.618, p<0.001).

**Fig 5:**
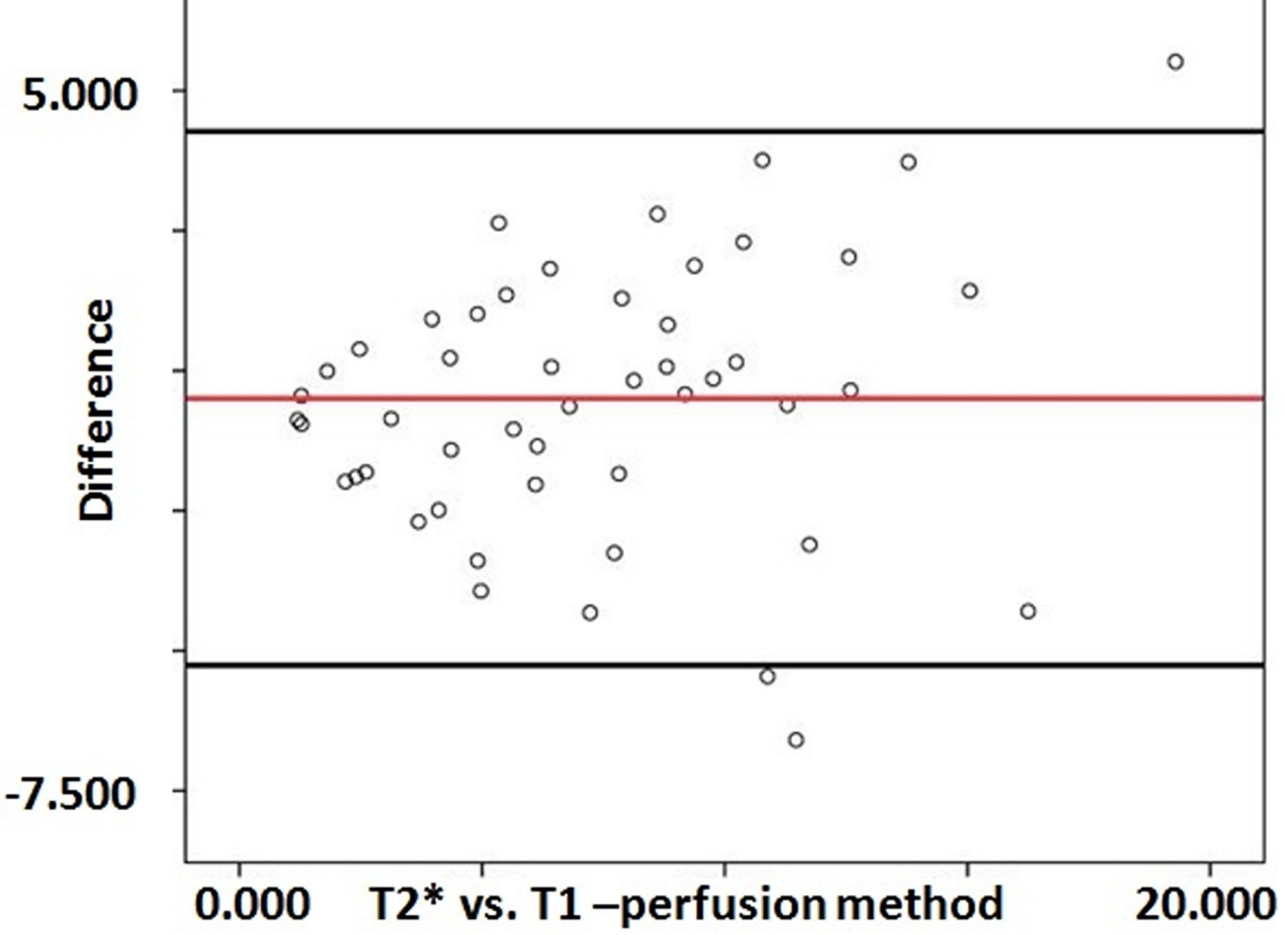
Bland-Altman is demonstrating the limits of agreements between the rCBV values recorded from two methods (T2*- and T1- perfusion MRI). The midline represents the mean values for the data points, and the top and bottom line represents the 1.96 and -1.96 limits of agreements, respectively.

The inter-technique agreement for maximum rCBV measurements on T2*- and T1-perfusion maps were good and showed an ICC value of 0.74 (p<0.001). Bland-Altman plots showed a good agreement between the rCBV values obtained using these two methods i.e. T2*- and T1- perfusion (Fig 5).

**Fig 6:**
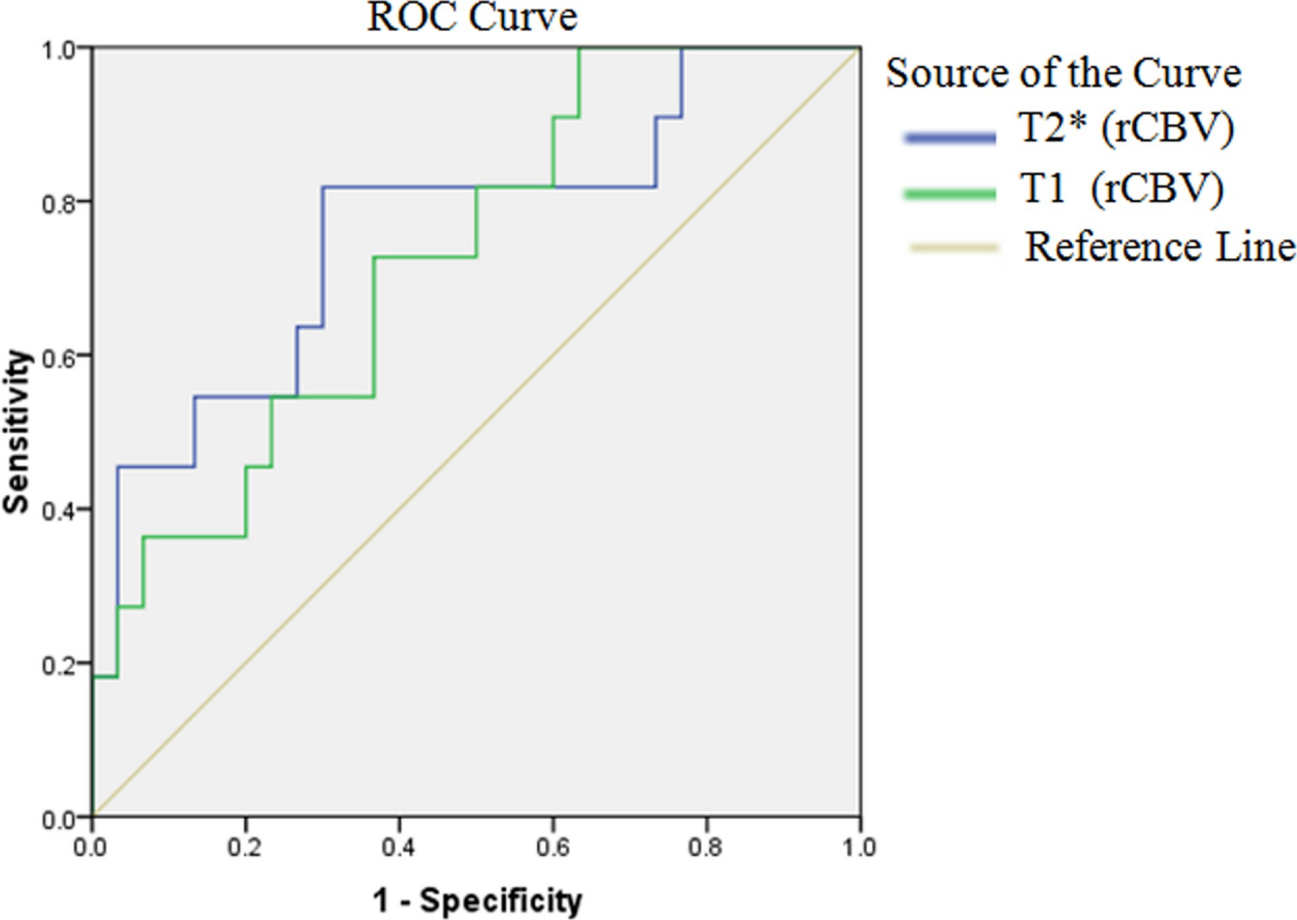
The Receiver operating characteristic (ROC) curves for the rCBV values derived from T1- and T2*-perfusion.

Comparison of grade-III and grade-IV tumors showed significantly higher rCBV values in grade-IV gliomas. The rCBV values derived from T2*- and T1-perfusion showed an AUC of 0.767; p = 0.040 and 0.723; p = 0.026; respectively for discrimination of grade-III and grade-IV gliomas. T2*-perfusion measured the rCBV cutoff value of 4.73 could discriminate grade-III from grade-IV gliomas with sensitivity and specificity of 81% and 72%; respectively. While T1-perfusion derived, rCBV showed a cutoff value of 5.47 discriminating grade-III and IV gliomas. It could discriminate the two tumor grades with a sensitivity and specificity of 78% and 63%; respectively (Table 2). Comparison of the ROC curves of T2*-perfusion measured rCBV and T1-perfusion measured rCBV for discriminating grade-III, and grade-IV gliomas did not show any statistically significant differences (Fig 6 and Table 2).

## Discussion

In this study, we compared the rCBV values derived independently using T1- and T2*-perfusion MRI in the patients with gliomas, and both techniques showed a good agreement with the comparable diagnostic performance for the discrimination of grade III and grade IV glioma. T2*-perfusion data was suboptimal in 32.63% of the patients due to the presence of significant intratumoral susceptibility arising from blood products, adjacent bone or sinuses in case of tumors located near to the skull base or post-surgical changes; on the other hand, all these tumors could be evaluated entirely by using T1-perfusion MRI.

As previously reported by other investigators that the T2*-perfusion data has some inherent limitations and it is more susceptible to the magnetic field inhomogeneity. On the other hand, T1-perfusion is less sensitive to the magnetic field inhomogeneity compared to T2*-perfusion and could be advantageous for brain tumor assessment especially in cases where T2*-perfusion images are not interpretable. However, limitations of T1-perfusion include a low temporal resolution, limited coverage, and lack of commercially available post-processing tools. Estimation of hemodynamic parameters using T1-perfusion method is considered problematic because the signal changes induced by the intravascular component of the tracer have T1-relaxivity in order of the magnitude smaller than for T2* images, and the small blood volumes in brain tissue do not allow for significant tissue concentrations [11].

In our study, we noted the higher number of suboptimal T2*-perfusion scans as compared to some of the previous studies [9,30,31]. One possible reason for the higher number of suboptimal scans is the inclusion of both pre and post-treatment studies. Another reason may be that ours was a retrospective study and hence this discrepancy may also arise due to the bias of reporting radiologist who would have preferred performing both T1- and T2*-perfusion after noting significant susceptibility on gradient images.

Few studies in the past have compared the T1- and T2*-perfusion-based methods for glioma evaluation have used the hemodynamic parameters derived from T2*-perfusion and tracer kinetic parameters (Volume transfer coefficient (Ktrans), fraction of EES (Ve), fraction of plasma volume (Vp) from T1- perfusion data. CBV estimation using T1-perfusion method is less common because of low temporal resolution and the signal changes induced by the intravascular component of the tracer have T1-relaxivity in order of magnitude smaller than for T2* image, and the small blood volumes in brain tissue do not allow for significant tissue concentrations [11]. In previous T1-perfusion studies, Vp has been used as a surrogate marker of angiogenesis and hemodynamic information [25,32] and has been shown to have a significant correlation with the rCBV values derived from the T2*-perfusion. However, some other studies have reported no correlation between the T1-perfusion derived Vp, and T2*-perfusion derived rCBV [33]. Also, Vp estimate may vary depending on the model applied and by the amount of contrast extravasation happening due to tumor leakage. This argues in favor of directly estimating the rCBV from the T1-perfusion as was done in the current study. Falk et al. reported rCBV derived from both methods in patients of glioma and the mean values of rCBV reported by authors were higher using T2*-MRI [23]. Larsen et al. showed that T1-perfusion method is useful for identifying neoplastic pathology in post-operative cases [34]. T1 perfusion data based kinetic parameters, particularly Ktrans and Ve, have been used previously for glioma gradings [27, 35, 36]. These parameters showed statistically significant difference beteen low and high grade glioms. However, these parameters performed poorly for differentiating grade III from grade IV tumors [35, 36].

Similarly, Santarosa et al. reported improved diagnostic performance of T1-perfusion method for brain tumor evaluation especially in the presence of susceptibility within the tumor [25]. Reviewer confidence has been found to be higher for the identification of the hot-spots on T1-perfusion derived Vp maps as compared to the T2*-perfusion derived rCBV maps, and results of the T1-perfusion could be replicated across multiple centers as well as multiple imaging platforms [32]. Similar to our observations, the authors reported the cases where no area of increased perfusion could be reliably identified in hemorrhagic lesions on T2*-perfusion map while T1-perfusion derived Vp could identify an abnormality in these lesions [9,32]. The observation that both techniques were comparable despite 1/3^rd^ of the T2*-perfusion scans being suboptimal, possible because most of the lesions were large and had enough non-hemorrhagic areas where no susceptibility changes were noted, and perfusion could be assessed with confidence using T2*-perfusion which in turn led to correct grade information. However, it becomes essential when assessing small lesions; planning image-guided biopsy of lesions and preoperative assessment as the whole tumor needs to be assessed to identify areas of increased perfusion correctly.

A significant limitation of the current study is the inclusion of a relatively small number of patients. In the current perfusion study, the T1-perfusion technique has limited coverage that is likely to be addressed in the future by using techniques like compressed sensing which can improve both temporal and spatial resolution [34]. Another caveat may be that even when T2*-perfusion data is not able to assess the entire tumor due to susceptibility artifacts, the presence of focally increased perfusion in tumor regions with no susceptibility may allow correct grade assessment. With the improved implementation, T1-perfusion technique with a single dose of contrast could be used to substitute T2*-perfusion method to overcome the limitations of applying this method in brain tumors and allow reliable perfusion measurements.

## Conclusion

We conclude that T1-perfusion with a single dose of contrast could be used as an alternative to T2*-perfusion to overcome the issues associated with this technique in brain tumors for reliable perfusion quantification. This is especially true for hemorrhagic, and post-treatment follow-up cases of gliomas.

## Abbreviations

BBB: Blood brain barrier
DCE: T1- perfusion (Dynamic contrast enhanced)
DSC: T2*-perfusion (Dynamic susceptibility contrast)
GBM: Glioblastoma multiforme
MRI: Magnetic resonance imaging
rCBV: Relative cerebral blood volume
